# Testicular seminoma clinical stage 1: treatment outcome on a routine care level

**DOI:** 10.1007/s00432-016-2162-z

**Published:** 2016-04-26

**Authors:** Klaus-Peter Dieckmann, Inken Dralle-Filiz, Cord Matthies, Julia Heinzelbecker, Jens Bedke, Jörg Ellinger, Petra Anheuser, Rainer Souchon, Uwe Pichlmeier, German Testicular Cancer Study Group

**Affiliations:** 1 Department of Urology, Albertinen-Krankenhaus Hamburg, Suentelstr. 11a, 22457 Hamburg, Germany; 2 Department of Urology, Bundeswehr Krankenhaus Hamburg, Lesserstr. 180, 22049 Hamburg, Germany; 3 Urologische Universitätsklinik, Universität des Saarlandes, Kirrberger Str. 1, 66424 Homburg/Saar, Germany; 4 Urologische Universitätsklinik Tuebingen, Hoppe Seyler Str. 3, 72076 Tuebingen, Germany; 5 Urologische Universitätsklinik Bonn, Sigmund Freud Str. 25, 53127 Bonn, Germany; 6 Universitätsklinik für Radioonkologie Tuebingen, Hoppe Seyler Str. 3, 72076 Tuebingen, Germany; 7 Institute of Epidemiology and Medical Statistics, Universitätsklinikum Eppendorf Hamburg, Martinistr. 52, 20251 Hamburg, Germany

**Keywords:** Testicular seminoma, Carboplatin, Radiotherapy, Surveillance, Rete testis

## Abstract

**Purpose:**

Clinical stage 1 (CS1) testicular seminoma involves an almost 100 % disease-specific survival in controlled clinical trials. We aimed to find out whether these results can be matched in patients managed on the routine care level.

**Patients, methods:**

In total, 725 patients with seminoma CS1 were prospectively enrolled from 130 institutions. Adjuvant management as decided by local physicians involved surveillance (*n* = 256), radiotherapy (41), 1× Carboplatin (362), and 2× Carboplatin (66). We registered type of management, age, duration of follow-up (F/U), relapse, rete testis invasion (RTI), and tumor size. Actuarial relapse-free survival curves were calculated for treatment modalities and stratified for tumor sizes and RTI. A Cox regression model was calculated to explore for factors influencing relapses.

**Results:**

Disease-specific survival was 100 %. Crude relapse rates were 8.2, 2.4, 5.0, and 1.5 % for surveillance, radiotherapy, 1× Carboplatin, and 2× Carboplatin after a median F/U of 30 months. RTI and tumor size were not associated with progression in surveillance patients. One course Carboplatin caused relapses in 6.8 % in tumor sizes >4 cm and 9.3 % (actuarial 13 %) in sizes >5 cm. The Cox model revealed the association of tumor size with recurrence in the entire seminoma population (Hazard ratio 1.17; 95 % confidence intervals 1.03–1.33).

**Conclusions:**

The overall outcome of CS1 seminoma managed on the routine care level mirrors that of controlled trials. Unexpectedly, the risk factors in surveillance patients were not confirmed, but tumor size proved to be a risk indicator in the entire group of seminoma. Importantly, one course Carboplatin involved low efficacy to control the disease in large tumors.

**Electronic supplementary material:**

The online version of this article (doi:10.1007/s00432-016-2162-z) contains supplementary material, which is available to authorized users.

## Introduction

Clinical stage 1 (CS1) seminoma represents the most frequent way of presentation of testicular germ cell tumors (GCTs) (Sokoloff et al. 2007; Heinzelbecker et al. 2011). The overall survival rate of nearly 100 % can be achieved by three management options as recommended in international guidelines (Albers et al. 2015; Oldenburg et al. 2013; Motzer et al. 2015). Currently, an expectant strategy with curative chemotherapy or radiotherapy to be applied only in the case of relapse is favored by European guidelines (Zengerling et al. 2013). A second option is adjuvant treatment with one course of Carboplatin AUC7 in all of the cases. A third alternative is the risk-adapted strategy with adjuvant Carboplatin therapy in the presence of risk factors and watchful waiting in the absence of these factors (Aparicio et al. 2014; Beyer et al. 2013). In this setting, a tumor size of greater than 4 cm and the invasion of seminoma cells into the rete testis represent recognized indicators for progression (Warde et al. 2002). However, these factors have been a matter of concern ever since their introduction into clinical practice. In fact, no prospective validation study has been conducted so far. Particularly, rete testis invasion (RTI) lacked evidence in several studies (Ruf et al. 2013; Soper et al. 2014), while the significance of tumor size was supported repeatedly (Mortensen et al. 2014; Chung et al. 2015). When Carboplatin was introduced into the treatment of seminoma, the standard regimen consisted of two courses of the drug to be applied with 4 weeks apart (Oliver et al. 1990; Dieckmann et al. 1996). One course of Carboplatin was considered sufficient according to a prospective randomized trial comparing abdominal radiotherapy with one course of Carboplatin where no inferiority of the drug regimen was found (Oliver et al. 2005). No formal comparative study has ever been conducted to prove the advantage of one course Carboplatin over the traditional two course regimen. Likewise, only three evaluations have supported the safety of the single shot treatment to date (Tandstad et al. 2011; Chau et al. 2015; Diminutto et al. 2015).

The present evaluation is a pattern of care analysis of CS1 seminoma patients in Germany (Dieckmann 2009). The first part of this project (National Seminoma Registry Study; NSR Study) had revealed surveillance to be currently the most frequently adopted management of seminoma patients (45 %) followed by the one course Carboplatin chemotherapy (35 %). Radiotherapy and the two courses regimen of Carboplatin are applied in less than 10 % (Dieckmann et al. 2016). Here, we report the results of follow-up of the patients registered to the study. We evaluated the relapse rates in the four management modalities, and we also looked to the significance of risk factors with respect to disease progression.

## Patients, methods

The principal aim of the present study was to analyze the treatment results in seminoma CS1 obtained in clinical routine practice (i.e., outside of clinical studies) with the treatment modalities that were endorsed by guidelines at the time of the study onset (Krege et al. 2008) and to compare these results with the data of high-standard controlled trials. During 2008–2013, a total of 1050 patients with testicular seminoma CS1 were registered from 130 institutions in Germany (online resource 1). Informed consent was obtained from all individual participants included in the study. Ethical approval was given by Ärztekammer Hamburg (PV 3123). Follow-up (F/U) information is available in 725 patients, and this subcohort represents the database of the present analysis. The treatment decisions were solely made at the discretion of local institutions with no influence from study regulations. The quantitative distribution of the treatment groups thus reflects the current clinical practice in this country (Dieckmann et al. 2016). We registered the following parameters in each patient: age (years), mode of management, size of the primary tumor (cm), RTI (yes/no), time-point during F/U (months), relapse (yes/no), and unexpected event (specify). The primary endpoint of this study was the frequency of relapse in the four treatment modalities. The secondary endpoint was the association of the factors tumor size and RTI with relapse in the various treatment modalities.

Follow-up examinations were performed by local urologists according to current guidelines (Hartmann et al. 2011). Results of the examinations were documented in a patient logbook with a copy of each report sent to the study center (Dieckmann 2009). In case of relapse, treatment was applied according to guidelines by local institutions (Krege et al. 2008).

All of the data were prospectively filed in a commercially available data base system (MS Excel). The final statistical analyses were performed with the SAS software package (version 9.3, SAS Institute, Inc., Cary, NC, USA) on a Windows platform. Actuarial relapse rates of the four treatment modalities were calculated with generating Kaplan–Meier curves. The log-rank test was used to compare the relapse rates relating to the treatment methods. To explore the role of tumor size and RTI, a stratified analysis was separately performed on the groups with surveillance and 1× Carboplatin, respectively, by analyzing relapse rates in various subgroups. Also, a multivariate stratified analysis was performed to look for combined effects of tumor size and RTI. Finally, a multivariate Cox regression model was calculated to establish Hazard ratios (HR) with 95 % confidence intervals (CIs) for relapse regarding the type of treatment, patient´s age, tumor size, and RTI. The chi-square test was used to compare Hazard ratios of the variables tested.

## Results

**Fig. 1:**
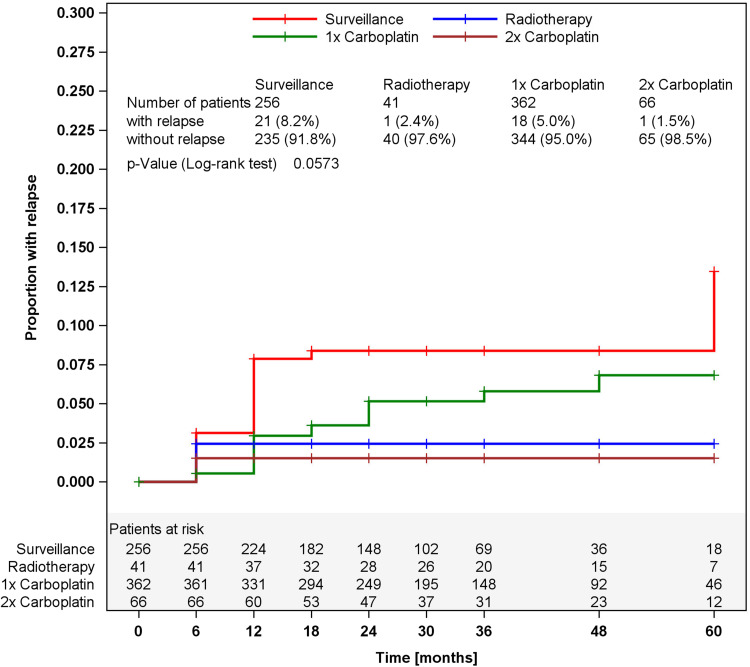
Time course of relapses in the four treatment modalities, Kaplan–Meier estimates

Of the 725 eligible patients, 256 were managed by surveillance, 41 with radiotherapy (20 Gy), 362 with one course Carboplatin AUC7, and 66 with 2 courses Carboplatin. The median duration of F/U is 30 months (6–60 months). Clinical details are listed in Table 1. A total of 41 relapses were noted, and all except one relapse were detected upon F/U by imaging methods. Anatomically, 39 relapses were located in the retroperitoneum, and each one in the lungs, and bone. For treatment of relapsing disease, 8 patients received curative radiotherapy, 28 cisplatin-based chemotherapy, and two chemotherapy with additional surgery. In 3 patients, the treatment of relapse is unknown (for more details, see online resource 2). All recurrences were cured generating a disease-specific survival rate of 100 %. Two patients (0.3 %) succumbed to unrelated events (1 cardiac event, 1 s malignancy). The relative proportions of recurrences in the treatment modalities are listed in Table 2. Kaplan–Meier curves showing the temporal course of the recurrences in each of the treatment modalities are provided in Fig. 1. The crude incidences of relapses among the four treatments are different though not statistically significant (log-rank, *p* = 0.0573). The majority of relapses developed within the first 2 years after primary treatment, but additional events occurred during the later course with the latest arising after 60 months in the surveillance group.

**Table 1:** Clinical details of the patients

	(*n*)	Age (years)		Tumor size (cm)		RTI
		Median	Range	Median	>4 cm	
Surveillance	256	40	20–75	2.5	25.3 %	17.5 %
Radiotherapy	41	39	25–65	4.4	68.3 %	7.4 %
Carboplatin 1×	362	42	19–82	4.0	49.0 %	65.8 %
Carboplatin 2×	66	43.5	21–81	4.0	50.0 %	9.3 %
*p*		0.86		<0.0001		<0.0001

Kruskal–Wallis test for comparison of ages and tumor sizes

Chi-square test for the presence of rete testis invasion (RTI), surveillance is the reference for comparison of groups

**Table 2:** Relapse rates and duration of follow-up in the four treatment groups

	Surveillance	Radiotherapy	1× Carboplatin	2× Carboplatin	Total
	*n* = 256	*n* = 41	*n* = 362	*n* = 66	*n* = 725
Relapse (*n*)	21	1	18	1	41
Rate	8.2 %	2.4 %	5.0 %	1.5 %	5.7 %
95 % CI	4.9–12.7 %	0.1–13.8 %	2.9–8.1 %	0–8.8 %	4.0–7.3 %
DWD	0	0	2 (0.6 %)	0	2 (0.3 %)
Median F/U (months)	24	36	30	30	30
Range of F/U (months)	6–60	6–60	0–60	6–60	0–60
*p* = 0.0573^a^					

*DWD* dead without disease, *F/U* follow-up

^a^Log-rank test for homogeneity of F/U duration

**Fig. 2:**
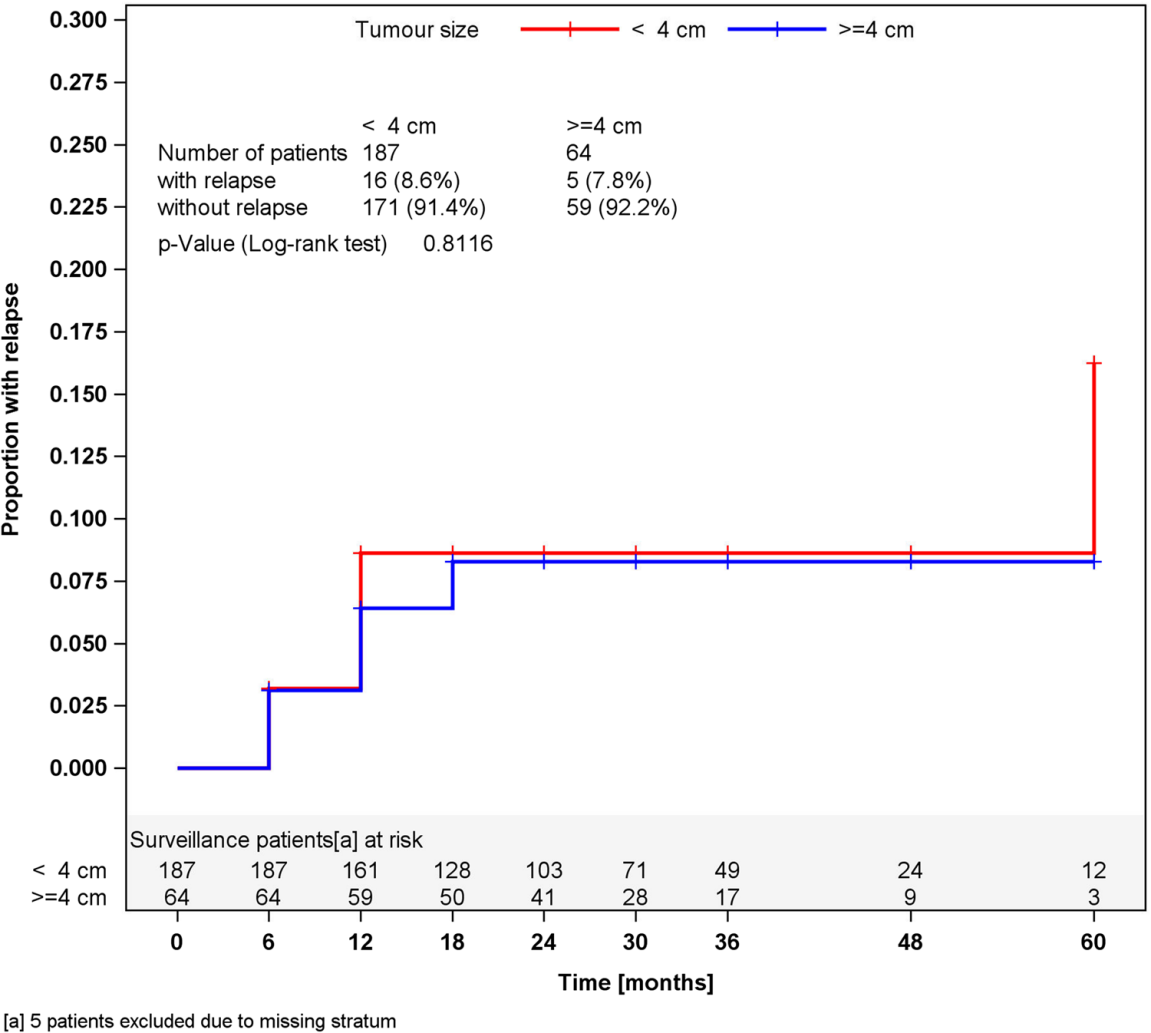
Time course of relapses in surveillance patients, stratified for tumor size, Kaplan–Meier estimates

**Fig. 3:**
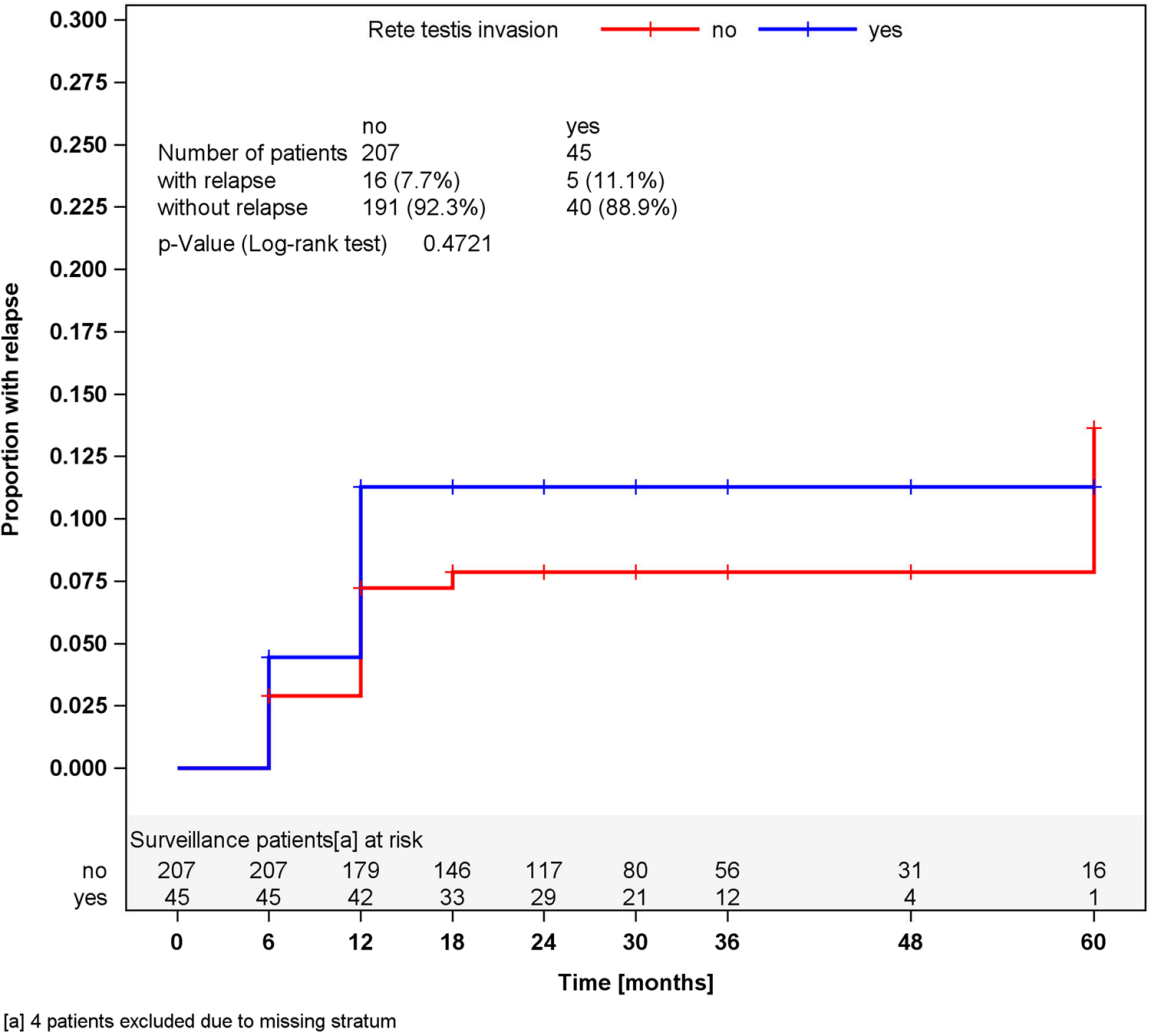
Time course of relapses in surveillance patients, stratified for rete testis invasion, Kaplan–Meier estimates

**Fig. 4:**
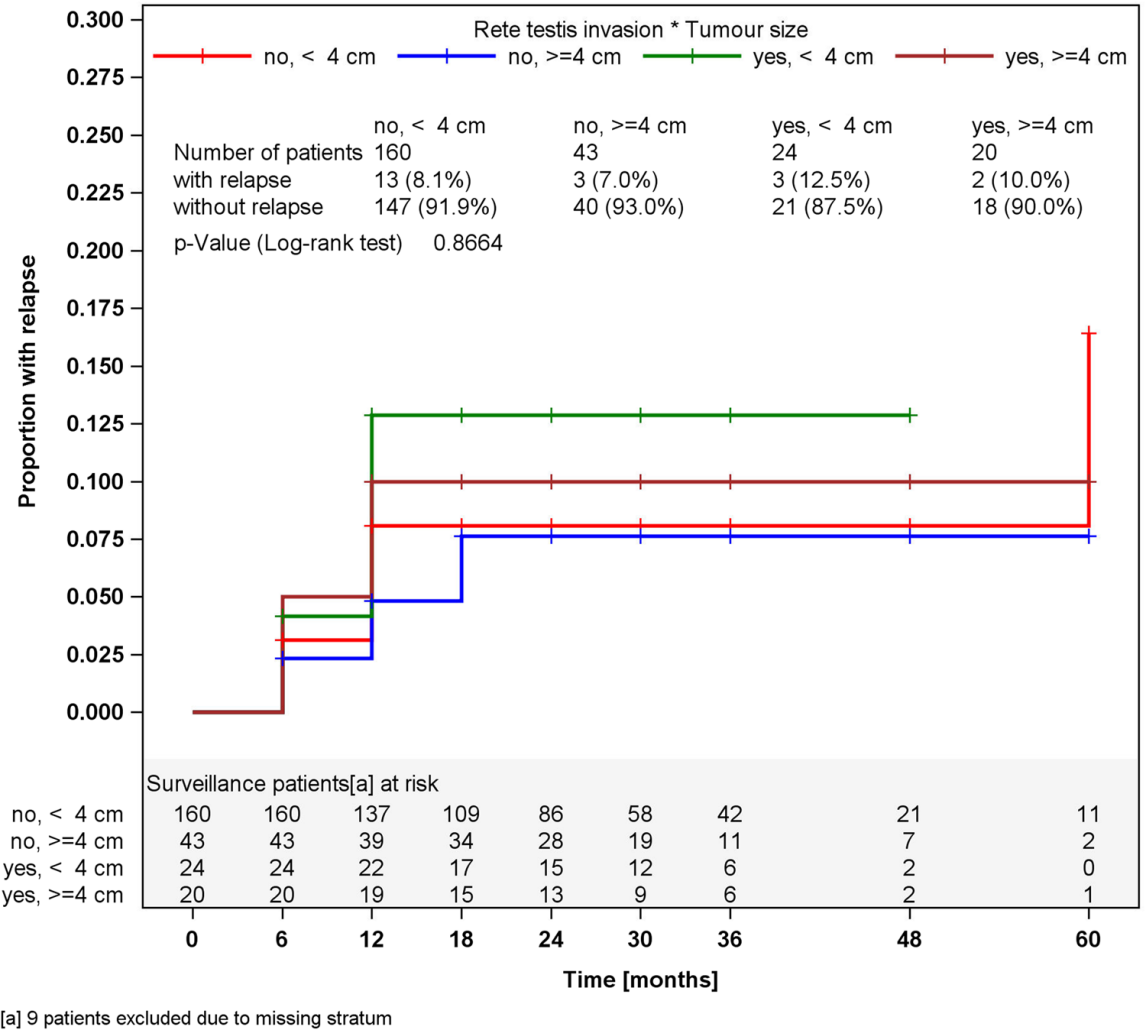
Time course of relapses in surveillance patients, stratified for rete testis invasion and tumor size, Kaplan–Meier estimates

**Fig. 5:**
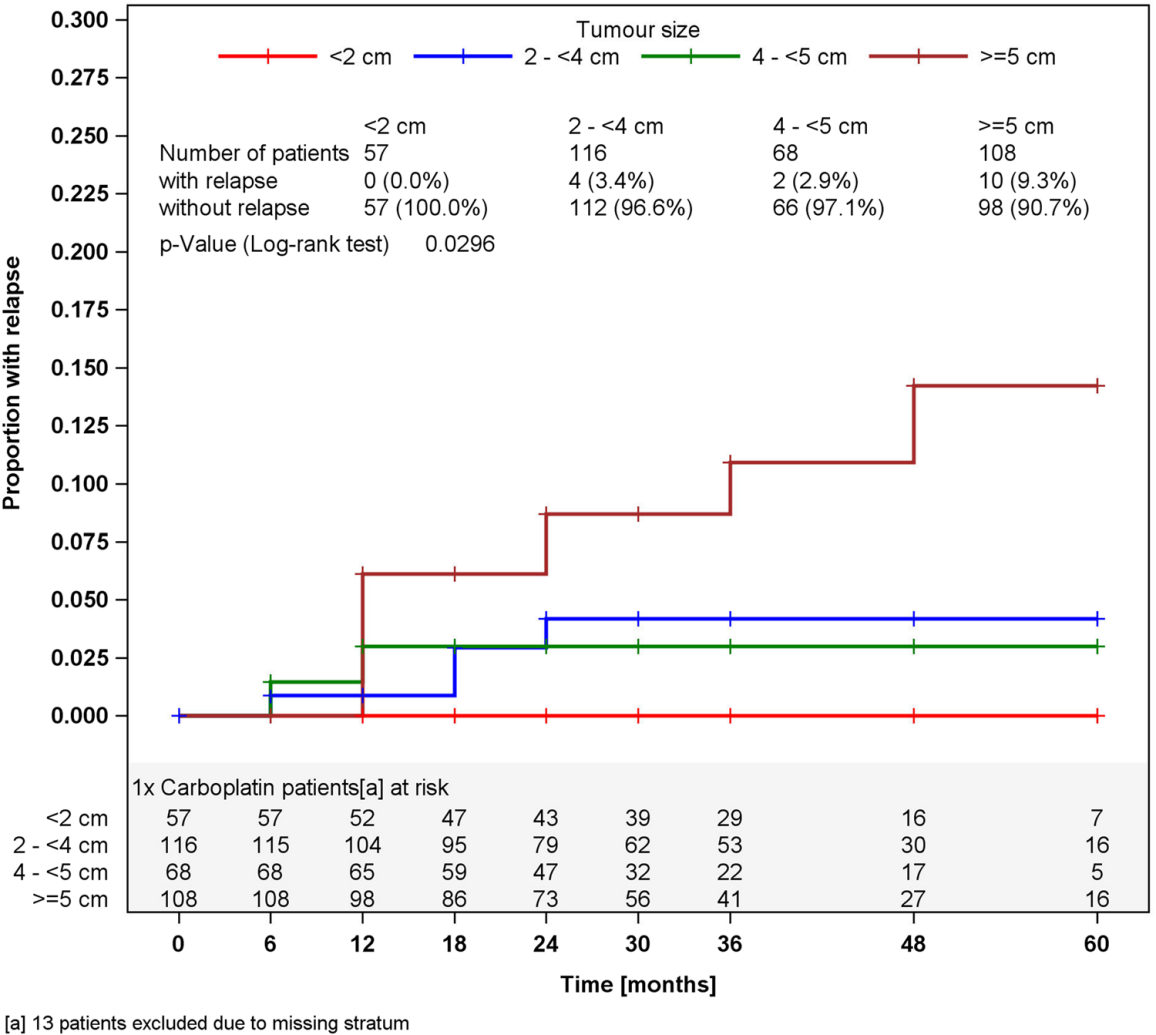
Time course of relapses in patients with one course Carboplatin, stratified for tumor sizes, Kaplan–Meier estimates

In surveillance patients, stratified analyses for tumor size of greater or less than 4 cm and for RTI, respectively, did not reveal different relapses rates in the strata (Figs. 2, 3). Accordingly, a multivariate stratified analysis regarding the combined effect of tumor size and RTI failed to reveal any predictive value of these factors with respect to relapse (Fig. 4). By contrast, among patients undergoing one course of Carboplatin, tumor size of greater than 4 cm was significantly associated (*p* = 0.0447) with relapse. Patients with tumors smaller than 4 cm relapsed in 2.3 % of cases (4 of 176) whereas 6.8 % (12 of 176) did so among those with tumors larger than 4 cm, resulting in a Hazard ratio of 3.03 (95 % CI 0.97–9.44) with respect to the progression risk of patients with tumors >4 cm. Further stratification of tumor size (Fig. 5) revealed that small tumors (<2 cm) had no relapse to date whereas those greater than 5 cm developed 9.3 % recurrences featuring an actuarial relapse rate of more than 13 % (*p* = 0.0296). Accordingly, relapse rates ranged around 3 % in the intermediate size categories. RTI was not associated with recurrence in this group (*p* = 0.1483). In a multivariate stratified analysis, looking for the combined effect of tumor size and RTI in the Carboplatin group, the highest relapse rate was found in patients with the two factors (9.1 %), but differences between subgroups were not significantly different.

Looking for factors influencing the recurrence of disease in the entire patient group, the Cox regression model adjusted for treatment mode revealed that size of the primary tumor involves a significantly increased Hazard rate of relapse of HR = 3.38 (95 % CI 1.10–10.41; *p* = 0.0338).

## Discussion

This study yielded five main results: (1) the overall treatment results of seminoma CS1 are excellent on the level of clinical routine practice, (2) surveillance strategies can be used successfully in this setting, (3) rete testis invasion and tumor size failed as indicators for progression in surveillance patients, (4) the one course Carboplatin regimen is less effective than expected, and (5) tumor size is predictive of relapse in patients receiving one course Carboplatin.

With respect to the primary endpoint of the present study, a 100 % disease-specific survival rate was observed in seminoma CS1. Thus, the overall therapeutic outcome achieved in routine practice is identical with that reported from high-standard controlled trials (Mortensen et al. 2014; Cummins et al. 2010; Aparicio et al. 2014; Kollmannsberger et al. 2015; Oliver et al. 2011; Soper et al. 2014; Hallemeier et al. 2014).

Accordingly, the surveillance strategy proved to be safe on the routine practice level. Only 21 relapses (8.2 %) occurred in this cohort. This markedly low rate may be related to the low prevalence of the risk factors RTI (17.5 %) and tumor size >4 cm (25 %) in this cohort. Obviously, the majority of the surveillance patients had been selected for this management because of the absent risk indicators. In view of the low prevalence of these factors in our cohort, the relapse rate of 8.2 % compares well with the rates of 8.3 % (Aparicio et al. 2014), 9.5 % (Ondrusova et al. 2015), and 12.2 % (Warde et al. 2002) reported for patients without risk factors. However, another reason for the comparatively low relapse rate is probably the rather short median observation time of 24 months in this cohort. As 75 % of the relapses arise during the first 2 years of surveillance (Kollmannsberger et al. 2015; Mortensen et al. 2014; Soper et al. 2014), one may expect more recurrences to come in a longer observation period, which is also highlighted by the Kaplan–Meier estimate of relapses of 13.5 % in our surveillance group.

It is of note that the recognized risk factors RTI and tumor size did not correlate with relapse in the surveillance cohort. However, selection bias could have contributed to this result because only less than one quarter of the patients in this subgroup had these factors. Nonetheless, those having one or two of these factors did not relapse more frequently than those without. Our report is thus in line with other series that failed to confirm RTI as a risk factor (Ruf et al. 2013; Chung et al. 2015).

Tumor size was not confirmed as a risk factor for progression in our surveillance cohort, and the same result was reported from a Japanese series (Kamba et al. 2010). Even so, this factor appears to be useful for predicting progression because the majority of previous studies had confirmed its validity (Chung et al. 2015; Mortensen et al. 2014; Aparicio et al. 2014; Ruf et al. 2013) and, moreover, tumor size qualified as a risk marker even in patients undergoing adjuvant therapy (Cohn-Cedermark et al. 2015; Chau et al. 2015).

In the cohort with one course of Carboplatin, 18 relapses (5 %, 95 % CI 2.9–8.1 %) occurred and this result is in line with the previously reported rates of 5.1 % (Oliver et al. 2011), 3.9 % (Tandstad et al. 2011), 4.0 % (Chau et al. 2015), and 5.2 (Diminutto et al. 2015) in non-risk-adapted series. With two courses of Carboplatin, we observed a relapse rate of only 1.5 % (95 % CI 0–8.8 %). Although at first glance, two courses appear to be more efficacious than one course, the relapse rates of the two regimens are not statistically different (*p* = 0.2048). However possibly, the small sample size of our two course cohort (*n* = 66) could have prevented formal statistical significance. In fact, low relapse rates of 1.7 % (Steiner et al. 2011), 3.2 % (Aparicio et al. 2014), and 3.6 % (Koutsoukos et al. 2015) with two courses were reported recently, supporting the view that two courses of Carboplatin might be more efficacious than just one (Dieckmann et al. 2000).

Concerns regarding the effectivity of one course Carboplatin to eradicate micrometastatic seminoma is fueled by the unexpected high relapse rate of 9.3 % (Kaplan–Meier estimate 13.5 %) in the presence of tumor sizes of >5 cm. Although chance effects might have contributed to this result, this high recurrence rate is clearly inferior to the results obtained with adjuvant radiotherapy (Hallemeier et al. 2014) and with the two course Carboplatin treatment (Aparicio et al. 2014). Similar data have been reported from the Swenoteca trial, where relapses after 1× Carboplatin were detected in 2.7 % in the absence of risk factors and in as many as 9.4 % when one or two risk factors were present (Cohn-Cedermark et al. 2015). In a series from UK, tumor size as a continuous variable was significantly associated with the relapse rate after 1× Carboplatin although the relapse rate was only slightly increased to 5.9 % in the patients with tumors larger than 4 cm (Chau et al. 2015). Likewise, 12.5 % relapses occurred with this regimen in the presence of risk factors in a small Slovakian series (Ondrusova et al. 2015).

Limitations of the present study derive from the lack of information regarding the applied dosages of Carboplatin. As dosing according to renal function is critical in the single shot Carboplatin regimen and dose reductions of 10 % might considerably impact treatment outcome (Cathomas et al. 2014), some of the relapses of this study could have been caused by inadvertent underdosing of the drug. However, the effect of underdosing has been questioned recently (Diminutto et al. 2015). Moreover, the remarkably high relapse rate in the patients with large tumors does probably not solely relate to potential underdosing of Carboplatin because our results are statistically quite robust and support for the findings comes from other studies (Cohn-Cedermark et al. 2015; Ondrusova et al. 2015). One other limitation of the study relates to the lack of F/U information in 325 of the 1050 patients originally enrolled. This markedly high proportion of lost patients may partly result from non-compliance of patients which is a well-known problem in young patients with GCT (Yu et al. 2009). Moreover, the lack of F/U information may also result from the German health system created fact that follow-up of patients is usually conducted by office urologists working independently of the institutions where the primary treatment was done. According to the particular design of the study as a pattern of care analysis with no rigid trial regulations, the study center had only minimal influence on participating institutions and almost none on the office urologists performing the F/U examinations.

As shown in the Cox regression model, tumor size of >4 cm represents a risk factor not only in patients under surveillance but also in those undergoing single shot Carboplatin therapy. In light of a 25 % relapse rate usually experienced in patients with tumor size >4 cm under surveillance (Warde et al. 2002), one course of Carboplatin reduced the anticipated relapse rates only by one half. This treatment is thus far less effective than expected from the pivotal studies (Oliver et al. 2011; Tandstad et al. 2011) and less effective than other adjuvant treatment modalities. The limited efficacy of Carboplatin in patients with large tumor sizes represents new knowledge that needs to be taken into account by upcoming guidelines. In practical terms, a plea could be made for applying two courses of the drug in patients with large tumors.

## Electronic supplementary material

Below is the link to the electronic supplementary material.

Supplementary material 1 (PDF 105 kb)

Supplementary material 2 (PDF 56 kb)
