# Supplementary material for: Testicular seminoma clinical stage 1: treatment outcome on a routine care level
Source: J Cancer Res Clin Oncol. 2016 Apr 26;142(7):1599–607. doi: 10.1007/s00432-016-2162-z (PMC4899489; doi:10.1007/s00432-016-2162-z)
Supplement: Supplementary file 1 — Supplementary material 1 (PDF 105 kb) [file 432_2016_2162_MOESM1_ESM.pdf]

Online resource 1:
Contributing Institutions ranked by the number of cases enrolled
Hamburg, Bundeswehr Krankenhaus
Hamburg, Albertinen-Krankenhaus
Tuebingen, Urologische Universitätsklinik
Trier, Krankenhaus der Barmherzigen Brüder
Mannheim, Urologische Universitätsklinik
Lohne, St. Franziskus Krankenhaus
Bonn, Urologische Universitätsklinik
Siegen, Jung-Stilling Krankenhaus
Ahaus, St. Marien Krankenhaus
Chemnitz, Zeisigwaldkliniken
Leipzig, St. Elisabeth Krankenhaus
Berlin, St. Hedwig Krankenhaus
Freiburg, Universitätsklinik
Karlsruhe, Städtisches Klinikum
Gladbeck, St. Barbara Hospital
Bergisch Gladbach, Marien-Krankenhaus
Köln, Heilig Geist Krankenhaus
Köln, Urologgia
Magdeburg, Universitätsklinik
Münster, Universitätsklinik
Reichenbach, Paracelsus-Klinik
Amberg, Klinikum St. Marien
Berlin, Bundeswehrkrankenhaus
Coburg, Städtisches Klinikum
Kaiserslautern, Westpfalzklinikum
Gütersloh, Städtisches Klinikum
München, Klinikum Bogenhausen
Ulm, Bundeswehrkrankenhaus
Würzburg, Julius Maximilian Universität
Frankfurt, Universitätsklinik
Fulda, Klinikum
Rüsselsheim, Klinikum
Schweinfurt, Leopoldina-Krankenhaus
Erlangen, Waldkrankenhaus St. Marien
Flensburg, Diako Klinikum
Frankfurt, Städtische Kliniken Höchst
Klinikum Ingolstadt
Köln, St. Elisabeth Krankenhaus
Lübbecke, Krankenhaus
Bad Tölz, Asklepios Stadtklinik
Hamm, St. Barbara Klinik
Herzberg, Urologie Herzberg

Jena, Urologische Universitätsklinik
Löningen, St. Anna Stift
Mönchengladbach, Maria Hilf Krankenhaus
Schwäbisch Hall, Diakonie Klinikum
Aachen, Urologische Universitätsklinik
Freiberg, Kreiskrankenhaus
Gera, Waldklinikum
Göttingen, Urologische Universitätsklinik
Geestland, Seepark Klinik
Plauen, Helios Klinikum
Aschersleben, Kreiskliniken
Berlin, Universitätsklinikum Charité
Bonn, Waldkrankenhaus
Erfurt, Krankenhaus St. Nepomuk
Hameln, Kreiskrankenhaus
Koblenz, Bundeswehrkrankenhaus
Lübeck, Universitätsklinikum Schleswig-Holstein
Reifenstein, Eichsfeld Klinikum
Wetzlar, Klinikum
Datteln, St. Vincenz Krankenhaus
Fürth, Klinikum
Garmisch-Partenkirchen, Klinikum
Gießen, Urologische Universitätsklink
Kempten, Klinikum
Köln, Urologische Universitätsklinik
Mannheim, Diakonie-Krankenhaus
Pforzheim, Siloah St. Trudpert Klinikum
Schwerin, Helios Kliniken
Stralsund, Helios Hanse Klinikum
Wilhelmshaven, Reinhard-Nieter-Krankenhaus
Bad Bergzabern, Klinikum Landau
Baden-Baden, Stadt-Krankenhaus
Bautzen, Oberlausitzkliniken
Berlin, Helios Kliniken
Berlin, Vivantes Auguste-Victoria-Krankenhaus
Dresden, Diakonissenkrankenhaus
Fürstenfeldbruck, Urologisches Zentrum
Hamm, St. Josef Krankenhaus
Homburg, Urologische Universitätsklinik
Münster, Herz-Jesu-Krankenhaus
Nürnberg, ÜGP
Solingen, Städtisches Klinikum
Ulm, Urologische Universitätsklinik
Augsburg, Klinikum
Bad Hersfeld, Klinikum

Bamberg, Klinikum Bruderwald
Bonn, Malteser Krankenhaus
Detmold, Klinikum Lippe
Frankfurt, Praxisklinik
Gehrden, Robert-Koch-Krankenhaus
Göttingen, Evangelisches Krankenhaus Weende
Hamburg, Praxisklinik
Hamburg, Praxisklinik
Hamburg, Universitätsklinikum Eppendorf
Hannover, Friederikenstift
Heidelberg, Krankenhaus Salem
Itzehoe, Klinikum
Lauchhammer, Klinikum Niederlausitz
Lüdenscheid, Märkische Kliniken
Magdeburg, Klinikum
Nürnberg, Theresien-KH
Rostock, Urologische Universitätsklinik
Schwedt, Asklepios Klinik
Stuttgart, Katharinenhospital
Würzburg, Juliusspital
Amberg, Praxisklinik
Bad Nauheim, Praxisklinik
Bitburg, Praxisklinik Dres. Zender/Müller
Bremen Praxisklinik am Klinikum Bremen-Nord
Bremen, Klinikum Bremen-Mitte
Deggendorf, Klinikum
Dessau, Diakonie-Krankenhaus
Frechen, St. Katharinen Krankenhaus
Hamburg, Asklepios Klinik Barmbek
Hamburg, Praxisklinik
Hannover, Siloah Krankenhaus
Herne, Marienhospital
Kempen, Hospital zum Heiligen Geist
Köln, Krankenhaus Holweide
Marktredwitz, Klinikum Fichtelgebirge
Memmingen, Klinikum
München, Urologische Universitätsklinik Großhadern
Pfullingen, Praxisklinik
Schwedt, Klinikum Uckermark
Weiden, Klinikum
Wesel, Marien-Hospital
Zirndorf, Praxisklinik
