# Supplementary material for: Testicular seminoma clinical stage 1: treatment outcome on a routine care level
Source: J Cancer Res Clin Oncol. 2016 Apr 26;142(7):1599–607. doi: 10.1007/s00432-016-2162-z (PMC4899489; doi:10.1007/s00432-016-2162-z)
Supplement: Supplementary file 2 — Supplementary material 2 (PDF 56 kb) [file 432_2016_2162_MOESM2_ESM.pdf]

Table (online resource 2) List of individual data of relapsing patients

#	Tumour size (cm)	RTI	treatment	Age at diagnosis (yrs)	Time to relapse (mon)	Localisation of relapse	Treatment for relapse
1	5.2	no	1 x Carbo	19	6	lungs	3 x PEB
2	2.4	no	1 x Carbo	42	12	retroperitoneal lymph nodes	radiotherapy
3	6.8	yes	1 x Carbo	34	18	retroperitoneal lymph nodes	PEB
4	6	yes	1 x Carbo	71	12	retroperitoneal lymph nodes	n/a
5	3.5	yes	1 x Carbo	46	18	retroperitoneal lymph nodes (bulky disease)	n/a
6	8	yes	1 x Carbo	48	12	inguinal lymph node	3 x PEB
7	4.5	yes	1 x Carbo	46	12	retroperitoneal lymph nodes ,13mm	3 x PEB
8	8	yes	1 x Carbo	49	9	retroperitoneal lymph nodes	3 x PEB
9	5.1	no	1 x Carbo	35	12	iliacal and retroperitoneal lymph nodes	3 x PEB+ pcRLA
10	2.7	no	1 x Carbo	47	24	retroperitoneal lymph nodes	3 x PEB
11	n/a	n/a	1 x Carbo	43	48	retroperitoneal lymph nodes	3 x PEB
12	4.5	n/a	1 x Carbo	34	12	retroperitoneal lymph nodes	3 x PEB
13	5.5	no	1 x Carbo	24	12	retroperitoneal lymph nodes	Radiotherapy 36 Gy
14	6	yes	1 x Carbo	52	12	iliacal lymph node, 2.1cm	Radiotherapy, 36 Gy
15	6	yes	1 x Carbo	26	24	retroperitoneal lymph node ,1.3cm	3 x PEB
16	n/a	yes	1 x Carbo	32	24	Iliacal lymph nodes	3 x PEB
17	3.8	yes	1 x Carbo	32	24	retroperitoneal lymph nodes	3 x PEB
18	7.5	yes	1 x Carbo	51	36	cervical lymph nodes	3 x PEB
19	12	yes	2 x Carbo	46	6	retroperitoneal and iliacal lymph nodes, 3.5 cm	3 x PEB
20	2.5	no	Surveillance	41	12	retroperitoneal lymph nodes	n/a

21	5.2	no	Surveillance	52	18	retroperitoneal lymph nodes	3 x PEB
22	4.6	no	Surveillance	46	12	retroperitoneal lymph nodes, 3.1cm	Chemotherapy
23	1.8	no	Surveillance	31	12	retroperitoneal lymph nodes, 129mm	3 x PEB
24	3.5	no	Surveillance	51	12	retroperitoneal lymph nodes	Radiotherapy 20 Gy (field)+ boost (involved node)
25	6	yes	Surveillance	50	12	retroperitoneal lymph nodes, 7 cm	3 x PEB
26	4.5	no	Surveillance	36	6	retroperitoneal lymph nodes	Radiotherapy 36 Gy
27	3	no	Surveillance	39	12	retroperitoneal lymph nodes, 2cm	3 x PEB
28	0.9	yes	Surveillance	52	6	retroperitoneal lymph nodes, 132mm	Radiotherapy 36 Gy
29	3	no	Surveillance	45	12	retroperitoneal lymph nodes	3 x PEB
30	2.4	no	Surveillance	46	6	retroperitoneal lymph nodes	3 x PEB
31	3	no	Surveillance	40	6	retroperitoneal lymph nodes 5cm	3 x PEB
32	3.6	no	Surveillance	26	12	retroperitoneal lymph nodes + pulmonal metastases	3 x PEB
33	2	no	Surveillance	40	6	retroperitoneal lymph nodes, 2cm	3 x PEB
34	4.5	yes	Surveillance	33	6	retroperitoneal lymph nodes	Radiotherapy 30 Gy
35	2.2	yes	Surveillance	26	12	retroperitoneal lymph nodes	3 x PEB
36	2	no	Surveillance	36	6	retroperitoneal lymph nodes	3 x PEB
37	1.2	no	Surveillance	33	48	retroperitoneal lymph nodes, 2.3cm	3 x PEB
38	1.5	yes	Surveillance	47	12	retroperitoneal lymph nodes	3 x PEB
39	3	no	Surveillance	41	12	retroperitoneal lymph nodes	Radiotherapy, 36 Gy
40	n/a	n/a	Surveillance	n/a	60	retroperitoneal lymph nodes	3 x PEB
41	n/a	n/a	radiotherapy	n/a	6	bone metastases	PEI chemotherapy plus surgery

n/a not available; PEB chemotherapy with Cisplatin, etoposide and Bleomycin; Carbo Carboplatin; pcRLA postchemo retroperitoneal lymph node dissection; yrs years; mon months; RTI rete testis invasion
